# Characteristics of initial symptoms in patients with dementia with Lewy body disease

**DOI:** 10.3389/fneur.2022.1024995

**Published:** 2022-10-12

**Authors:** Min Fei, Fei Wang, Hao Wu, Shuai Liu, Jinghuan Gan, Yong Ji

**Affiliations:** ^1^Clinical College of Neurology, Neurosurgery and Neurorehabilitation, Tianjin Medical University, Tianjin, China; ^2^Department of Neurology, Yuncheng Central Hospital, Shanxi Medical University, Yuncheng, China; ^3^Tianjin Key Laboratory of Cerebrovascular and Neurodegenerative Diseases, Department of Neurology, Tianjin Dementia Institute, Tianjin Huanhu Hospital, Tianjin, China; ^4^Department of Neurology, Beijing Tiantan Hospital, China National Clinical Research Center for Neurological Diseases, Capital Medical University, Beijing, China

**Keywords:** dementia with Lewy bodies, initial symptoms, gender differences, prevalence, age

## Abstract

**Background:**

Dementia with Lewy bodies (DLB) is the second most common neurodegenerative dementia. Although DLB is characterized by fluctuating cognitive impairment, some symptoms may appear before cognitive impairment, including rapid eye movement, sleep behavior disorder (RBD), psychiatric symptoms, autonomic symptoms, Parkinson's symptoms, etc. Therefore, DLB may be misdiagnosed as other diseases in its early stage.

**Objective:**

This study aimed to investigate the characteristics of initial symptoms of DLB, which could potentially offer essential clues for the earliest diagnosis of this disorder.

**Methods:**

A total of 239 patients with probable DLB who visited the cognitive impairment outpatient department of Tianjin Huanhu Hospital from September 2015 to March 2021 were consecutively enrolled. We retrospectively evaluated the initial symptoms of all included participants. The time of onset of initial symptoms was also assessed.

**Results:**

The most frequent initial symptom was memory loss (53.9%), followed by psychiatric symptoms (34.7%), RBD (20.9%), parkinsonism (15.1%), and autonomic symptoms (10.1%). Significant gender and age differences existed in the initial symptoms of patients with DLB.

**Conclusions:**

Our study elucidated the initial symptoms in patients with probable DLB. RBD was significantly more reported by men than by women, whereas women showed a higher incidence of visual and auditory hallucinations. A better understanding of the initial symptoms of DLB could lead to a more accurate diagnosis.

## Introduction

Dementia with Lewy body disease (DLB) is the second most common neurodegenerative dementia, accounting for approximately 10–15% of patients with dementia (1). DLB has a highly heterogeneous clinical course and overlaps with Parkinson's and Alzheimer's disease in clinical and pathological manifestations (2). The main pathological feature of DLB is that there are diffusely distributed Lewy bodies in the cerebral cortex and subcortical gray matter nuclei. Studies have confirmed that the damage of cholinergic and monoamine neurotransmitters in patients with DLB may be related to cognitive impairment and extrapyramidal motor impairment (2).

DLB is characterized by fluctuating cognitive impairment and other symptoms, including parkinsonism, rapid eye movement (REM), sleep behavior disorder (RBD), visual hallucinations, and autonomic symptoms (3). DLB has a similar impairment in memory, execution, and visual-spatial functions as AD. But at the early stage of cognitive impairment, it is easy to be confused when the accompanying symptoms are atypical. Amnestic cognitive impairment is the main manifestation of AD, while the amnestic symptoms of DLB are not prominent. In the initial stage of illness, patients with DLB may be expected to present with visual-spatial abnormalities and executive dysfunction, or more probably, a combination of these symptoms. The initial symptoms of DLB can occur up to decades before the diagnosis of dementia (4). The analysis of the early symptoms of DLB can be useful in clarifying the clinical features of DLB, preventing misdiagnoses, and avoiding inappropriate treatments. To date, reports of the initial symptoms have been limited.

This study retrospectively investigated the characteristics and timing of initial symptoms in 239 patients with probable DLB. They can provide important clues to anticipate the diagnosis of this disorder. We also investigated the relationship of age and gender with the initial symptoms.

## Materials and methods

### Participants

We retrospectively investigated 239 patients with probable DLB who consecutively attended the cognitive impairment outpatient department of Tianjin Huanhu Hospital from September 2015 to March 2021. The patients who were first visited in other departments, such as psychiatric outpatient and then transferred to our cognitive impairment outpatient department were included. The inclusion criteria were as follows: age between 40 and 90 years, clinical diagnosis of probable DLB, cooperation, and ability to complete evaluation scales. The diagnosis of DLB was performed in accordance with the diagnostic criteria developed by the DLB Consortium in 2005 and 2017. All patients presented with at least two core symptoms, one core symptom, and at least one suggestive feature. The exclusion criteria were as follows: diagnosis of Alzheimer's disease, vascular dementia, or other types of dementia; diagnosis of concomitant diseases of the nervous system, such as cerebrovascular diseases, brain infections, or epilepsy; diagnosis of depression or any other mental disorder; diagnosis of relevant concomitant disorders, including cardiopulmonary diseases, hematological diseases or other system diseases; inability to complete the assessments or refusal to participate in the research.

### Measures

Clinical features, dementia scales, and neuroimaging were assessed at the time of diagnosis. All subjects underwent clinical assessment by two trained neurologists. Computed tomography (CT) and magnetic resonance imaging (MRI) were used for neuroimaging. C-labeled Pittsburgh compound B positron emission tomography (^11^C-PIB PET) scans were used to indicate the pathological process of Aβ in the brain. ^18^F-fluorodeoxyglucose positron emission tomography (^18^F-FDG PET) scans were used to evaluate the metabolism of brain tissues in different regions.

We recorded the information of the following initial symptoms during an interview with patients and caregivers:

- Memory loss, including anterograde (inability to learn new memories) and retrograde (forgetting old memories);
- Parkinsonism, including resting tremor, muscular rigidity, bradykinesia, and postural instability;
- RBD and repeated episodes of sleep-related vocalization and/or complex motor behaviors during sleep;
- Psychiatric symptoms, including visual and auditory hallucinations, delusions, anxiety, depression, and apathy;
- Autonomic symptoms, including gastrointestinal symptoms (dysphagia, salivation, early abdominal fullness, constipation, looser stools, fecal incontinence), urinary symptoms (urinary urgency, urinary incontinence, incomplete emptying, weak stream of urine, frequency, nocturia), and cardiovascular symptoms (orthostatic hypotension, lightheaded when standing for some time, syncope).

We also retrospectively investigated the onset of initial symptoms. We introduced the time-to-dementia variable, corresponding to the time elapsed from the onset of symptoms to the diagnosis of dementia.

### Statistical analyzes

Data are expressed as absolute numbers, percentages, or mean ± standard deviation. The T-test was used when the data were normally distributed. The Mann–Whitney U-test was used for non-normal distributions. The chi-square test was used to compare categorical variables. All statistical tests were performed using SPSS version 26.0 (SPSS Inc., Chicago, US). Statistical significance was considered with a *P* < 0.05.

## Results

### Patient demographics and prevalence of initial symptoms

We retrospectively investigated 239 patients with probable DLB, including 120 (50.20%) men and 119 (49.80%) women. The mean age at onset was 67.76 ± 9.22 years. The mean age at the diagnosis was 71.67 ± 7.86 years. The average age of education was 9.55 ± 4.27 years. The average scores of the mini-mental state examination (MMSE) were 15.34 ± 6.96. Each patient reported more than one symptom. The most frequent initial symptom was memory loss (53.97%). Psychiatric symptoms were also frequent initial symptoms (34.70%), followed by RBD (20.92%), Parkinson's disease (15.06%), and autonomic nerve symptoms (10.04%).

### Gender differences in the initial symptoms of patients with DLB

**Figure 1:**
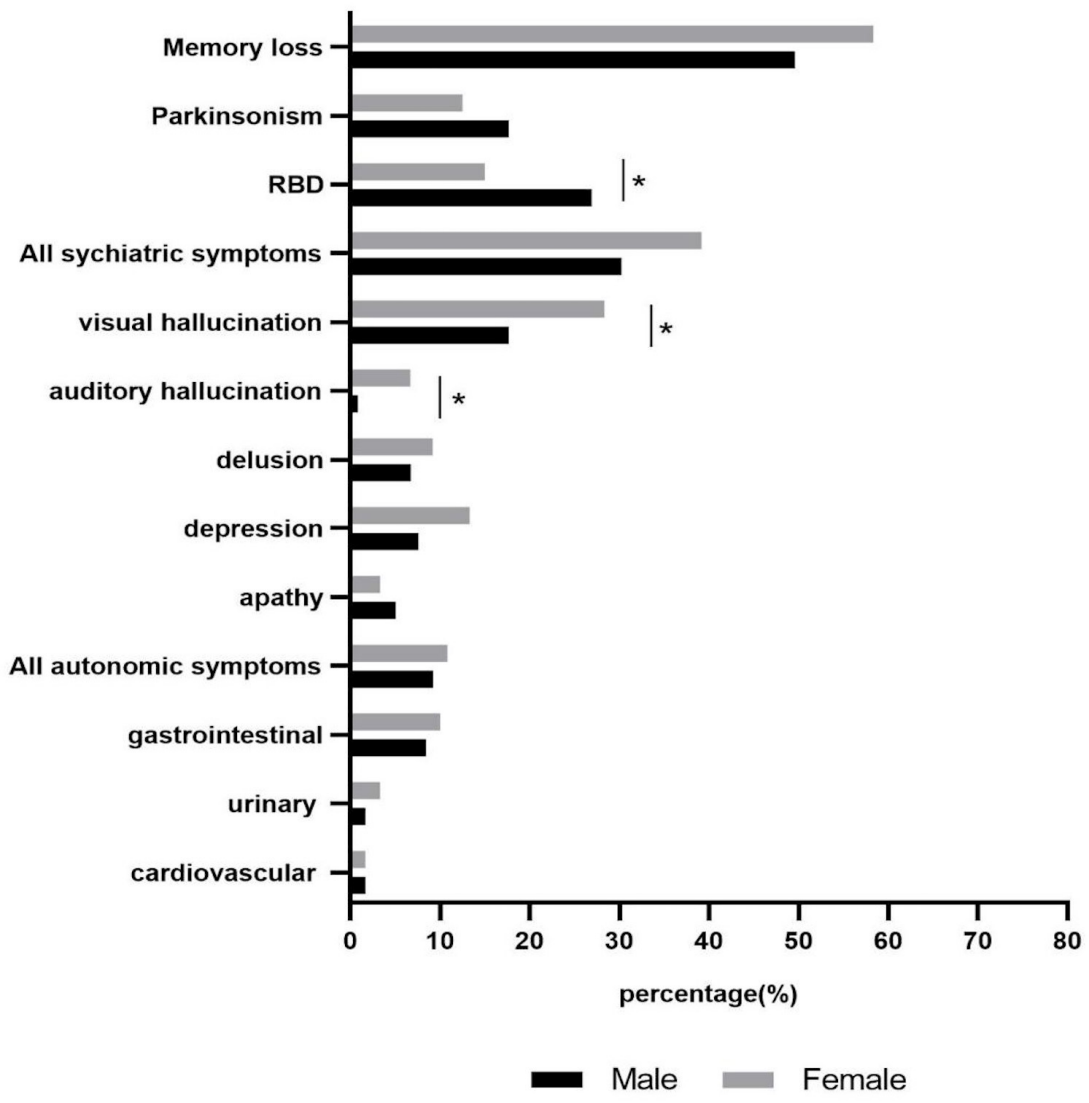
Gender differences in initial symptoms of patients with DLB. * The results showed significant differences between male and female. *p < 0.05.

Men reported the presence of RBD significantly more often than women (*p* = 0.024), while psychiatric symptoms were more common in women (men: 30.30%, women: 39.20%). Compared with men, women reported significantly more visual (*p* = 0.048) and auditory hallucinations (*p* = 0.036). Delusion, depression, and autonomic symptoms were more common in women than in men, without statistically significant differences. Apathy and parkinsonism were more common in men than women, without a statistically significant difference (Figure 1).

### Correlation between age and initial symptoms of patients with DLB

**Figure 2:**
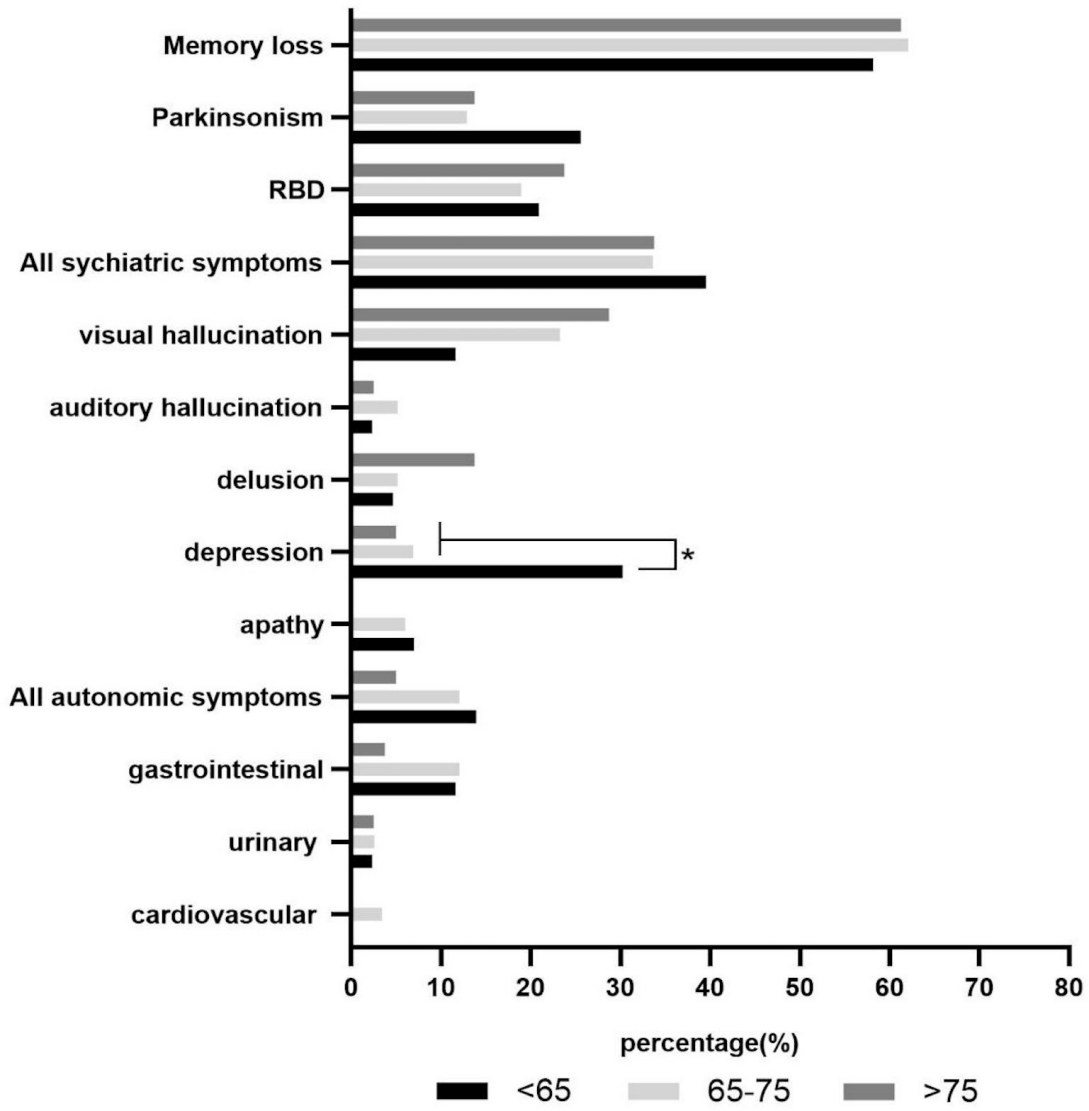
Correlation between age and initial symptoms of patients with DLB. * The results showed significant difference between middle-aged and elder patients. *p < 0.001.

The initial symptoms appeared between the ages of 32 and 88 years. They occurred in patients under 65 years, between 65 and 75 years, and over 75 years of age in 43 (17.99%), 116 (48.54%), and 80 (33.47%) patients, respectively. Middle-aged patients (<65 years old) showed higher rates of memory loss, parkinsonism, and psychiatric symptoms. Elderly patients (≥ 65 years old) reported memory loss, RBD, and psychiatric symptoms more frequently. There was a statistically significant difference in the incidence of depression between middle-aged and elderly patients (*p* < 0.001; Figure 2).

### The duration of initial symptoms before diagnosis

**Figure 3:**
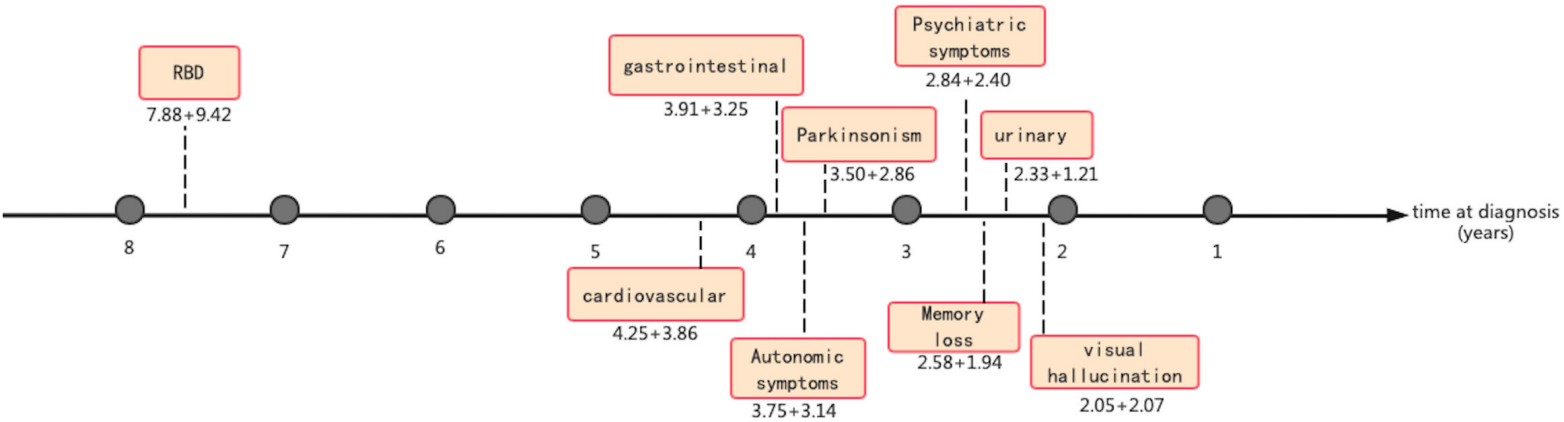
The duration of initial symptoms before diagnosis.

The duration of initial symptoms before diagnosis is reported (Table 1). The initial symptoms occurred on average 3.89±5.25 years before diagnosis. The average duration of memory loss, parkinsonism, RBD, psychiatric symptoms, and autonomic symptoms before diagnosis was 2.5 ± 1.94, 3.50 ± 2.86, 7.88 ± 9.42, 2.84 ± 2.40, and 3.75 ± 3.14 years. The visual hallucinations lasted for 2.05 ± 2.07 years on average (Figure 3).

**Table 1:** The duration of initial symptoms before diagnosis in DLB patients.

**Initial symptoms**	**duration before diagnosis (year)**
Meantime	3.89 ± 5.25
Memory loss	2.58 ± 1.94
Parkinsonism	3.50 ± 2.86
RBD	7.88 ± 9.42
Psychiatric symptoms	2.84 ± 2.40
Visual hallucination	2.05 ± 2.07
Auditory hallucination	2.22 ± 2.49
Delusion	2.16 ± 1.61
Depression	3.68 ± 3.02
Apathy	2.20 ± 1.32
Autonomic symptoms	3.75 ± 3.14
Gastrointestinal	3.91 ± 3.25
urinary	2.33 ± 1.21
cardiovascular	4.25 ± 3.86

## Discussion

DLB is the second most common dementia following Alzheimer's disease, characterized by the deposition of α-synuclein in Lewy bodies (5). Because the onset of the initial symptoms can occur 15 years or more before dementia onset, it is difficult to make a precise early diagnosis of DLB (6). There are limited reports on this topic. This study retrospectively investigated the prevalence and characteristics of initial symptoms in 239 patients with probable DLB.

A previous study reported the presence of several symptoms before the diagnosis, including cognitive decline, sleep disturbances, motor symptoms, and autonomic symptoms (7). In our study, memory loss was the most common initial symptom (53.97%), followed by psychiatric symptoms, RBD, parkinsonism, and autonomic symptoms in DLB. Another study found that female patients were more likely to present with psychotic and mood symptoms than males (8). In a cross-sectional survey, the cumulative and 1-month frequencies of visual hallucinations were 60.0 and 55.4% in women and 44.8 and 41.4% in men, respectively (9). These results agree with our study. We reported that the prevalence of visual and auditory hallucinations in women was significantly higher than in men. In addition, men reported higher rates of RBD than women. Another study demonstrated that the psychiatric manifestations of AD were associated with gender-specific tau phosphorylation abnormalities, and female patients showed significantly higher levels of phosphorylated tau (10). Considering the similar pathophysiological mechanisms of DLB and AD (11), we suggest that our gender difference may be due to biological differences between male and female patients with probable DLB. Auditory hallucinations were also common early symptoms in women with probable DLB, often accompanied by visual hallucinations. We found that women reported a higher rate of auditory hallucinations than men (6.67 vs. 0.84%). In accordance, Tsunoda et al. (12) described higher rates of auditory hallucinations in women with DLB. Utsumi et al. (13) showed that women experienced significantly more auditory hallucinations (8.3%) than men (1.0%). It is unclear why women with DLB are more likely to develop auditory hallucinations. The dopaminergic system might be involved in the occurrence of auditory hallucinations (14).

Depression is more common in DLB than in other types of dementia, including Alzheimer's disease (15). Depression occurs in approximately 33% of patients with DLB (16). We found that depression, as the initial symptom, was more common in middle-aged patients with probable DLB. Depression is listed as a supportive feature in the 2017 criteria for the clinical diagnosis of DLB (13). The literature has not reported a potential correlation between age and depression in patients with DLB. Panegyres et al. showed that patients with early-onset Alzheimer's disease reported more depression than patients with late-onset Alzheimer's disease (17). Some studies reported that patients with dementia and depression developed cognitive dysfunction earlier than nondepressed patients (18). Altogether, these findings suggest that the early onset of dementia is a risk factor for depression. However, the mechanisms remain unclear. We showed that depression was more common in women than in men without a statistically significant difference. The overall prevalence of depression is higher in women than in men because of personality and social factors (19). Other studies found no correlation between depression, dementia, patients' age, cognitive function, and ability to function daily (20, 21). Depression may occur at any time during DLB, with an interaction between age and gender (22).

Early symptoms appear several years before the diagnosis of DLB. According to a retrospective study, approximately 35% of patients showed cognitive dysfunction 1 year before diagnosis. Molano et al. (23) described an autopsy-confirmed DLB patient with recurrent visual impairment 3 years before the onset of cognitive dysfunction. Visual hallucinations preceded memory loss in 6.7% of patients (24). In our study, visual hallucinations lasted, on average, for only 2.05 years. It is necessary to consider DLB in elderly women who report visual hallucinations.

Recent studies suggest that RBD may be an early symptom of neurodegenerative disorders associated with synuclein deposition, such as DLB, Parkinson's disease, and MSA (25). RBD patients showed biomarkers consistent with synuclein diseases, and autopsies of RBD patients demonstrated an underlying synucleinopathy in most cases (26, 27). In patients with RBD, peripheral tissues such as the enteric nervous system also showed abnormal α-synuclein immunoreactivity (28, 29). These findings suggest that RBD is presumably associated with an underlying synucleinopathy. In this study, we found that RBD preceded probable DLB on average by 7.88 ± 9.42 years. RBD could be used as a predictor of probable DLB. In DLB patients, the incidence of RBD was up to 76%, anticipating the emergence of DLB by several years (30). Takayuki et al. showed a correlation between RBD and DLB (31). In early-onset DLB patients with RBD, Kasanuki K et al. found a significant deterioration of the dopaminergic pathways (32). In addition, a metabolic decrease was observed in the parietal lobe, prefrontal area, precuneus, central gyrus, and amygdala (33), suggesting that RBD might play a role in the pathogenesis of DLB.

This study has several limitations. The retrospective nature of this study may have resulted in unintentional inaccuracies in data collection and missing variables. We enrolled patients with probable DLB because of the lack of autopsy data. Moreover, the COVID-19 pandemic is suggested to have a negative impact on depression, anxiety, stress, and other mental health conditions, especially on women, teenagers, elderly people living alone, and people with mental disorders and other mental diseases. We did not consider the impact of the COVID-19 pandemic on the psychology of DLB patients, which is the limit of this article.

## Conclusions

In conclusion, this is the first study conducted in China to explore the initial symptoms of patients with probable DLB. Gender differences have been described, suggesting that gender stratification should be fully considered in clinical trials and research of new therapies in the future. More attention should be focused on screening for mental symptoms in early-onset DLB patients, especially depression. As an early predictor of DLB, DLB patients with RBD may have faster cognitive impairment and autonomic nerve dysfunction. Effective intervention by RBD could ameliorate the development of DLB.

## Data availability statement

The raw data supporting the conclusions of this article will be made available by the authors, without undue reservation.

## Ethics statement

The studies involving human participants were reviewed and approved by the Committee for Medical Research Ethics at Tianjin Huanhu Hospital and the Tianjin Health Bureau. The patients/participants provided their written informed consent to participate in this study. Written informed consent was obtained from the individual(s) for the publication of any potentially identifiable images or data included in this article.

## Author contributions

MF: writing the initial draft, design of methodology, investigation, and revising the manuscript. FW: analyzing data, investigating, and revising the manuscript. SL: investigation, visualization, and interpretation of data. HW: investigation and data management of the center. JG: investigation, project administration, and revising the manuscript. YJ: design of the study, funding acquisition, supervision, and revising the manuscript. All authors contributed to the article and approved the submitted version.

## Funding

This work was supported by the National Natural Science Foundation of China (Grant No. 82171182), Science and Technology Project of Tianjin Municipal Health Committee (Grant No. ZC20121 and KJ20048), and Tianjin Key Medical Discipline (Specialty) Construction Project (No. TJYXZDXK-052B).

## Conflict of interest

The authors declare that the research was conducted in the absence of any commercial or financial relationships that could be construed as a potential conflict of interest.

## Publisher's note

All claims expressed in this article are solely those of the authors and do not necessarily represent those of their affiliated organizations, or those of the publisher, the editors and the reviewers. Any product that may be evaluated in this article, or claim that may be made by its manufacturer, is not guaranteed or endorsed by the publisher.
