# Supplementary material for: Characteristics of initial symptoms in patients with dementia with Lewy body disease
Source: Front Neurol. 2022 Oct 12;13:1024995. doi: 10.3389/fneur.2022.1024995 (PMC9596794; doi:10.3389/fneur.2022.1024995)
Supplement: Supplementary file 1 [file Data_Sheet_1.docx]

Gender differences in initial symptoms of patients with DLB

| Initial symptoms | All（n=239） | Male(n=119） | Female（n=120） | χ^2^ | P-value |
| --- | --- | --- | --- | --- | --- |
| Memory loss | 129(53.97%） | 59（49.58%） | 70（58.33%） | 1.552 | 0.213 |
| Parkinsonism | 36(15.06%） | 21（17.65%） | 15（12.50%） | 1.637 | 0.201 |
| RBD | 50(20.92%） | 32（26.89%） | 18（15.00%） | 5.106 | 0.024 |
| Psychiatric symptoms | 83(34.72%） | 36（30.25%） | 47（39.17%） | 2.095 | 0.148 |
| visual hallucination | 55(23.01%) | 21 (17.65%） | 34（28.33%） | 3.851 | 0.048 |
| auditory hallucination | 9(3.77%) | 1（0.84%） | 8（6.67%） | - | 0.036 |
| delusion | 19(7.95%) | 8（6.72%） | 11(9.17%) | 0.488 | 0.405 |
| depression | 25(10.46%) | 9（7.56%） | 16(13.33%) | 2.124 | 0.145 |
| apathy | 10(4.18%) | 6(5.04%) | 4(3.33%) | 0.113 | 0.736 |
| Autonomic symptoms | 24(10.04%） | 11（9.24%） | 13（10.83%） | 0.167 | 0.683 |
| gastrointestinal | 22(9.21%) | 10（8.40%） | 12（10.00%） | 0.182 | 0.669 |
| urinary | 6(2.51%) | 2（1.68%） | 4（3.33%） | - | 0.684 |
| cardiovascular | 4(1.67%) | 2（1.68%） | 2（1.67%） | - | 1.000 |

Correlation between age and initial symptoms of patients with DLB

| Initial symptoms | <65（n=43) | 65-75（n=116） | >75（n=80） | F/χ^2^ | P-value |
| --- | --- | --- | --- | --- | --- |
| Memory loss | 25（58.14%） | 72(62.07%） | 49(61.25%） | 0.205 | 0.922 |
| Parkinsonism | 11(25.58%） | 15(12.93%） | 11(13.75%） | 4.112 | 0.128 |
| RBD | 9(20.93%） | 22(18.97%） | 19（23.75%） | 0.655 | 0.721 |
| Psychiatric symptoms | 17（39.53%） | 39（33.62%） | 27（33.75%） | 0.535 | 0.765 |
| visual hallucination | 5（11.63%） | 27（23.28%） | 23（28.75%） | 4.637 | 0.096 |
| auditory hallucination | 1（2.32%） | 6（5.17%） | 2（2.50%） | 0.946 | 0.661 |
| delusion | 2（4.65%） | 6（5.17%） | 11（13.75%） | 3.254 | 0.227 |
| depression | 13（30.23%） | 8（6.90%） | 4（5.00%） | 18.151 | 0.000^a,b^ |
| apathy | 3（6.98%） | 7（6.03%） | 0（0%） | 2.051 | 0.395 |
| Autonomic symptoms | 6（13.95%） | 14（12.07%） | 4（5.00%） | 3.867 | 0.167 |
| gastrointestinal | 5（11.63%） | 14（12.07%） | 3（3.75%） | 4.925 | 0.085 |
| urinary | 1(2.32%） | 3（2.59%） | 2（2.50%） | 0.232 | 1.000 |
| cardiovascular | 0(0%） | 4（3.45%） | 0（0%） | 2.955 | 0.179 |

^a^<65 vs 65-75; ^b^ <65 vs >75

# The duration of initial symptoms prior to diagnosis in DLB patients

| Initial symptoms | duration prior to diagnosis (year) |
| --- | --- |
| Mean time | 3.89+5.25 |
| Memory loss | 2.58+1.94 |
| Parkinsonism | 3.50+2.86 |
| RBD | 7.88+9.42 |
| Psychiatric symptoms | 2.84+2.40 |
| visual hallucination | 2.05+2.07 |
| auditory hallucination | 2.22+2.49 |
| delusion | 2.16+1.61 |
| Depression | 3.68+3.02 |
| apathy | 2.20+1.32 |
| Autonomic symptoms | 3.75+3.14 |
| gastrointestinal | 3.91+3.25 |
| urinary | 2.33+1.21 |
| cardiovascular | 4.25+3.86 |
